# Dynamic Carbon-Neutrality Assessment Needed to Tackle the Impacts of Global Crises

**DOI:** 10.1021/acs.est.2c04412

**Published:** 2022-07-08

**Authors:** Peng Jiang, Christian Sonne, Siming You

**Affiliations:** † Department of Industrial Engineering and Management, Business School, Sichuan University, Chengdu 610064, China; ‡ Department of Ecoscience, Arctic Research Centre, Aarhus University, Frederiksborgvej 399, DK-4000 Roskilde, Denmark; § James Watt School of Engineering, University of Glasgow, University Avenue, Glasgow G12 8QQ, U.K.

**Keywords:** carbon neutrality, global warming, emissions

Ambitious carbon neutrality plans are required to achieve the 1.5 °C goals of global warming mitigation under the 2016 Paris Agreement. For instance, compared with the business-as-usual case without any policy intervention, China needs to reduce carbon emissions by more than 90% and cut energy consumption by over 39% to achieve the 1.5 °C goals, which would cost 2.8–5.7% of its gross domestic product (GDP) by 2050.^1^ The European Union (EU) has been active in carbon reduction for decades. A recent study showed that 78% of 327 studied cities in the EU aimed to reduce carbon emissions by 47%, but these cities need to double their efforts by 2050 to reach the goals of the 2016 Paris Agreement.^2^ These actions toward carbon neutrality are challenging, but the global league is continuing its efforts. New global collaboration and decarbonization pathways are being formulated as exemplified by the agreement of a 30% cut in methane emissions by 2030 signed by more than 100 countries.^3^

Unfortunately, global security events such as (a) regional wars, (b) the COVID-19 pandemic, (c) cyberattacks, and (d) large-scale wildfires are threatening the gains towards reaching the carbon neutrality goals. The existing carbon neutrality action plans were formulated without considering the influences of emerging crises, which have the potential to trigger a series of new energy or environmental issues. Specifically,

(a) In response to the war in Ukraine, the European Commission published a communication about a joint European action in March 2022 for more secure, affordable, and sustainable energy, such as liquefied natural gas, biomethane, and renewable hydrogen, to reach independence for Russian fossil fuels before 2030. The corresponding REPowerEU Plan on energy saving, supplier diversification, and accelerated transition of renewable energy was released by the European Commission on May 18, 2022. The spatiotemporal consequences of the joint European action to the fulfilment of carbon neutrality goals have not been evaluated comprehensively. The fast transition will not be an easy one-shot thing due to the nontrivial impacts of the uncertainties in renewable energy resources in the EU. The ban on 90% of Russian crude by the end of 2022 agreed by the EU leaders triggers a new wave of oil price surge. The price hikes add immediate pressure on the EU and global consumers, and incur inflation and economic instability, which will, in turn, increase the costs of the transition to renewable energy. It is expected that the transition costs will increase further by the end of 2022 due to Russia’s banning of various types of materials (e.g., neon, krypton, and xenon) that are essential for making chips.
(b) Upon global economic recovery from the long-lasting COVID-19 pandemic, 15.3 gigatons of coal consumption-related CO_2_ were emitted in 2021 alone. This accounted for more than 40% of the overall growth in the emissions, being the highest in history.^4^ Despite the fact that the lockdown and quarantine measures worldwide reduced the global emissions in 2020 slightly, newly induced structural changes add to postpandemic uncertainty. These include for example behavioral preference for coal consumption, the private car preference worldwide and the desire for economic recovery that will backlash the emissions gain in the longer run.
(c) The International Energy Agency highlighted that cybersecurity threats would become one of the major challenges against the increasing adoption of cleaner energy with the recent emergence of cyberattacks on wind and solar projects. However, companies often do not have coping experiences or sufficient prevention plans when implementing green transition and digital transformation. The year 2021 experienced a record-breaking number of cyberattacks. With the development of more sophisticated cyber systems in the energy sector, cyberattacks-inflicted system breakdown will hinder the steady progress of the green transition and the carbon neutrality ambition.
(d) The occurrence of large-scale wildfires becomes more frequent, partially because of global climate change. The European Space Agency claimed that the wildfires worldwide destroyed about four million square kilometers of Earth’s land each year, being equal to about half the size of the United States. Large-scale wildfires, in turn, contribute to greenhouse gas (GHG) emissions and global warming, aggregating the situation while reducing available resources for biofuel production and thereby the achievement of carbon neutrality.

Here we appeal a dynamic carbon-neutrality assessment framework explored by the environmental communities and respond to the crises and their uncertainties. The United Nations (UN) reviews and revises its near-term national mitigation targets every five years, which benefits the construction of the dynamic assessment framework on the global scale. However, the increasing emerging crises warrant similar efforts for a fine-grained region given that the current COVID-19 pandemic and Ukraine war are both resulting in spatiotemporal heterogeneity in energy intensity and resource imports/exports. A recent study based on near-real-time carbon emission analysis shows that CO_2_ emissions in 2021 consume 8.7% of the remaining carbon budget under the 2016 Paris Agreement, and the budget could be used up 9.5 years from now.^5^ That implies the urgency of developing a dynamic assessment framework in a shorter time frame.

Considering the demands in both the spatial and temporal dimensions, it is essential to redesign the roadmaps of ambitious carbon neutrality goals using a dynamic assessment framework:

I. On a tactical level, different regions/countries with carbon neutrality commitments should develop specific scenario-based solutions with the approach of life cycle assessment (LCA). The scenarios incorporate the projection of emerging crises based on the understanding of existing crises. The uncertainties of the global security events necessitate scenario-based consequential LCA in a shorter period. Informed by the tactical solutions, a new carbon neutrality management mode can be developed to achieve greater levels of preparedness and resilience.
II. On a strategic level, central organizations like the UN need to design a two-scale coupling mechanism to coordinate the tactical solutions across different regions/countries with different levels and types of socio-economic, technical, and environmental needs. Different tactical solutions on the region/country scale focus on their own input–output efficiency if no incentives and regulations from a third party are provided, resulting in local optima only. The coupling mechanism targeting a global optimum should be incorporated into UN’s quinquennial review and revision of nationally determined contributions on a global scale.

We appeal to rethink the carbon neutrality ambition and its connection with the existing and emerging global crises. It is believed that better carbon-reduction practices can be developed by coherently integrating the dynamic assessment framework with the development of new-generation technologies like carbon capture, efficient energy storage, and renewable energy innovation.
